# The Conditional Effect of Scientific Knowledge and Gender on Support for COVID-19 Government Containment Policies in a Partisan America

**DOI:** 10.1017/S1743923X20000458

**Published:** 2020-07-09

**Authors:** Carlos Algara, Sam Fuller, Christopher Hare

**Affiliations:** 1 University of Texas at El Paso; 2 University of California, Davis; 3 University of California, Davis

**Keywords:** gender, latent scientific knowledge, COVID-19 pandemic, policy support, scaling

## Abstract

With the onset of the COVID-19 pandemic in the United States, many state and local governments were forced to implement necessary policies to contain transmission of the deadly virus. These policies ranged from closing most businesses to more controversial proposals, such as postponing primary elections. In this research note, we examine the role that scientific knowledge and gender played in citizen perceptions of these virus containment policies, both in the general population and among partisans. We find that while a gender gap persists in scientific knowledge, both in the general population and within the parties, women are generally more likely to use this knowledge to inform their policy views on necessary government action during the COVID-19 pandemic. These findings shed light on how knowledge and gender intersect to drive support for government intervention during the time of a severe public health crisis in a partisan America.

The onset of the coronavirus disease 2019 (COVID-19) pandemic revealed a distinct lack of preparation and decisive intervention by the world's governments. In the United States alone as of early July 2020, there were more than 2.67 million documented cases resulting in 125,000 deaths, increasing the need to prevent the spread of the virus. Despite the risks posed by COVID-19, the mass public's perceptions and beliefs about the virus and relevant policies are mixed. While both the federal government and states have implemented policies to contain the transmission of the virus by restricting various societal activities, little is known about what specifically informs support for or opposition to such interventions.

To better understand the determinants and influences on these policy preferences, this article assesses the relationship between gender, scientific knowledge, partisanship, and COVID- 19 policy attitudes. We find significant evidence of a gender gap in how scientific knowledge influences support for COVID-19 containment policies. Specifically, we find that women better incorporate scientific knowledge when generating informed opinions regarding COVID-19-related policies than men. This relationship is present among members of both parties.

## LINKING GENDER AND KNOWLEDGE IN SUPPORT FOR GOVERNMENT INTERVENTION

Previous research consistently identifies a gender gap between men and women's scientific knowledge and consequent support for and trust in science (Gauchat 2012; Miyake et al. 2010). Explanations for this gap tend to focus on differences in the educational and cultural expectations of men and women, especially the stereotype that women are worse at science than men (Miyake et al. 2010). Research in political science identifies a gender gap in men and women's policy preferences, with many differences persisting over decades (Lizotte 2020; Shapiro and Mahajan 1986). These gaps are concentrated, domestically, around crime and punishment, the environment, social welfare, and, most importantly, health care (Cochran and Sanders 2009; Schlesinger and Heldman 2001; Shapiro and Mahajan 1986). Various explanations offered for this gap between men and women are summarized and tested by Schlesinger and Heldman (2001).

The differences identified by Schlesinger and Heldman that are relevant to assessing support for COVID-19 containment policies are likely captured in the different perceptions that women have toward government interventions and their higher average level of compassion for others relative to men. Specifically, these authors find that women support prosocial policies because of their higher level of compassion and perception of higher government effectiveness than men. Other relevant research identifies the linkage between policy preferences and differences in risk perceptions and aversion (e.g., Huddy, Feldman, and Cassese 2009) and the influence of differing social roles and values between men and women (e.g., Diekman and Schneider 2010).^1^

Finally, recent work directly examines the motivations for women's support for health care provision more generally and the Affordable Care Act (ACA) specifically, finding that women support the ACA more than men in large part because of their higher level of prosocial values (Lizotte 2020). Furthermore, recent research on COVID-19 is consistent with this compassion and prosocial explanation for gender differences at the elite level. For example, Shay (2020) finds that female health commissioners and administrators were much more likely to adopt stay-at-home orders before their male counterparts.

All of this research supports the idea that the underlying mechanisms that inform policy preferences for women differ significantly than those for men. When women consider their support for COVID-19 containment policies, they are likely drawing on their prosocial considerations (possibly informed by their social roles), a general increased trust in government programs, and a reduction in the risk of harm for themselves and others. However, these considerations should only be considered by women if they *know* that COVID-19 is dangerous and has the potential to inflict great amounts of harm. Intuitively, the likelihood that any given individual should have this COVID-19 knowledge, we would expect, should be determined by their own level of general scientific knowledge, with greater levels increasing the probability that one understands the true gravity of COVID-19. Thus, we should expect that, all else being equal, women who possess high scientific knowledge should be more likely than men to support COVID-19 containment policies.

## PARTISAN VARIATION IN INTERACTIVE EFFECTS

Against the backdrop of a deeply fractured political system, characterized by intense partisan and ideological polarization over a range of issues and demographics (Gibson and Hare 2016), COVID-19 presents an uncommon instance of a salient issue on which voters have few, if any, existing considerations to guide their policy preferences. Policies addressing COVID-19 are not only politically unfamiliar but also represent highly technical, “hard” issues (Carmines and Stimson 1980) involving matters of medical and epidemiological expertise. Generally speaking, it is more difficult for voters to constrain their attitudes on hard issues along existing ideological lines (Pollock, Lilie, and Vittes 1993).

Under these conditions, public opinion should be more fluid and receptive to nonpolitical information flows (Zaller 1992). This is especially the case in situations involving anxiety and fear related to disease outbreaks and pandemics, which pushes individuals to be more likely to listen and heed the advice of medical or scientific experts (Albertson and Gadarian 2015). Hence, we expect that scientific literacy will play a larger role in shaping attitudes on COVID-19 policies than on sother scientific-based debates such as climate change (e.g., Kahan et al. 2012). However, these effects should also be conditioned by partisanship, as Republicans have received mixed, confusing, and conflicting cues from Republican elites and other right-leaning figures (Motta, Stecula, and Farhart 2020). In contrast, Democratic messaging has been consistent in its support for scientific experts and the need for significant interventions (Green et al. 2020). Indeed, Shay (2020) finds evidence that implementation of stay-at-home policies were more prevalent in states with Democratic governors. Consequently, we expect that scientific knowledge should play a more muted role in shaping Republicans’ policy preferences.

## RESEARCH DESIGN

To evaluate our hypotheses, we rely on nationally representative survey data provided by the Pew Research Center's American National Trends Panel Survey. Given potential concerns regarding endogeneity between COVID-19 attitudes and scientific knowledge, we rely on panel wave 32 fielded from January 7 to 21, 2020, to measure our key independent variable of citizen scientific literacy. To measure mass attitudes about COVID-19 policies, we rely on panel wave 64 fielded from March 19 to 24, 2020. In this survey, panelists were asked whether it was necessary for the government to restrict the following collective activities to contain COVID-19:

1. International travel
2. Most businesses (except grocery stores and pharmacies)
3. Large gatherings of more than 10 people
4. Major sporting and entertainment events
5. K–12 schooling
6. Restaurant dining
7. Upcoming state primary elections (i.e., to postpone due to the virus)

These outcome variables were coded 1 if citizens felt it was necessary for the government to restrict such activity and 0 if they felt it was unnecessary. We construct separate composite measures of COVID-19 policy preferences and latent scientific knowledge using item response theory (IRT) models.^2^ Lastly, to measure our conditioning variable of gender, we rely on self- reported gender identification.^3^

To test the conditional effects of knowledge and gender on support for intervention policies, we specify a logistic regression model for each of the seven outcome variables. We include an interaction term of knowledge and gender allowing us to estimate how the effect of scientific knowledge varies by gender. We also estimate a regression model evaluating the interactive effect of gender and scientific knowledge on our composite measure of policy preferences.

To provide a baseline comparison of scientific knowledge effects, we specify an additive model omitting the interaction terms. All of our models include the standard control variables of partisanship, ideology, age, education, race, income, and region.^4^ To explore variation in our effects of interest, we estimate models by both partisanship and on the full sample, providing a total of 24 models evaluated.

## RESULTS

**Figure 1.**
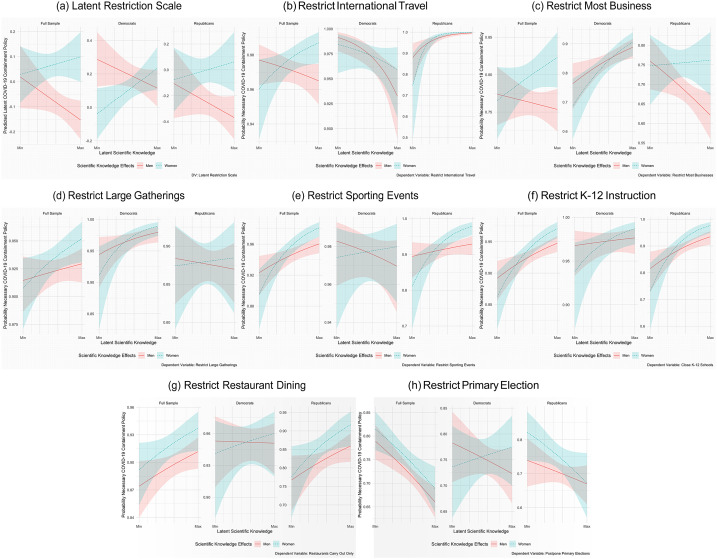
Interactive effects of scientific knowledge and gender by policy.

The results of our models for the full and party-specific samples can be found in Figure 1, which shows the effect of moving from the minimum to maximum value of scientific knowledge on policy support for each gender and the baseline. In all, we find a positive and significant effect of scientific knowledge on COVID-19 containment policies among women in five baseline, four Democratic, and four Republican models. In the regression model assessing the interactive effect of knowledge and gender on COVID-19 policy preferences, we find that knowledge significantly increased support among Democratic women.

Overall, although women in our sample have, consistent with previous research, lower average scientific knowledge than men, those who are more knowledgeable are much more likely to support COVID-19 interventions. We argue that women's higher trust in government programs and their compassion toward other individuals helps explain this gender gap in how scientific knowledge is used to inform policy preferences. This higher trust and compassion is only triggered, however, when women have requisite levels of scientific knowledge. While we do observe similar effects of scientific knowledge among men, they are much weaker. Furthermore, the impact of scientific knowledge on women persists across parties, with Republican women becoming much more supportive of interventions as their scientific knowledge increases compared to their male copartisans.

Importantly, we also find consistent support that scientific knowledge decreases the probability of deeming it necessary to postpone state primary elections. Indeed, these findings come on the heels of recent survey data showing strong bipartisan support for alternative modes of voting during the COVID-19 pandemic. Future work, both in terms of theory and empirical design, should assess the role scientific knowledge plays in informing both support for democratic institutions and accessibility to the ballot. Especially with respect to the full sample, we find consistent support that higher knowledge lowers the probability of supporting the postponement of elections.

## CONCLUSION AND FUTURE WORK

Taken together, these results suggest that women incorporate scientific knowledge into their preferences for COVID-19 interventions in a substantively different way than men. These results comport with previous findings on women's higher levels of compassion and prosocial preferences, perceptions of government program effectiveness, and higher perceptions of and aversion toward risk: when women possess the knowledge to understand the dangers of COVID-19, their support of COVID-19 containment policies dramatically increases. In all, our results contribute to our understanding of the underlying mechanisms that guide policy preference formation, especially in relation to gender, partisanship, and new issues.

## Footnotes

1. See Lizotte (2018) for a comprehensive summary of possible explanations for this gender gap in policy preferences.

2. Consistent with our expectations, COVID-19 restriction attitudes and liberal-conservative identification are only weakly correlated (ρ = 0.18), with liberals somewhat more likely to support restrictions. Details and validation of both IRT models are provided in the appendix.

3. This is measured on a binary scale by Pew Research Center, with only “refused” as an additional category.

4. The full model description and results can be found in the appendix. Models are specified with survey weights (*N* = 2,775).

## Supplementary material

For supplementary material accompanying this paper visit http://dx.doi.org/10.1017/S1743923X20000458. click here to view supplementary material
