# Supplementary material for: The Conditional Effect of Scientific Knowledge and Gender on Support for COVID-19 Government Containment Policies in a Partisan America
Source: Politics & Gender. 2020 Jul 9:1–9. doi: 10.1017/S1743923X20000458 (PMC7684197; doi:10.1017/S1743923X20000458)
Supplement: Supplementary file 1 [file S1743923X20000458sup001.pdf]

1 Supplemental Appendix

Figure 1
Differences in Latent Scientific Knowledge by Gender & Sample

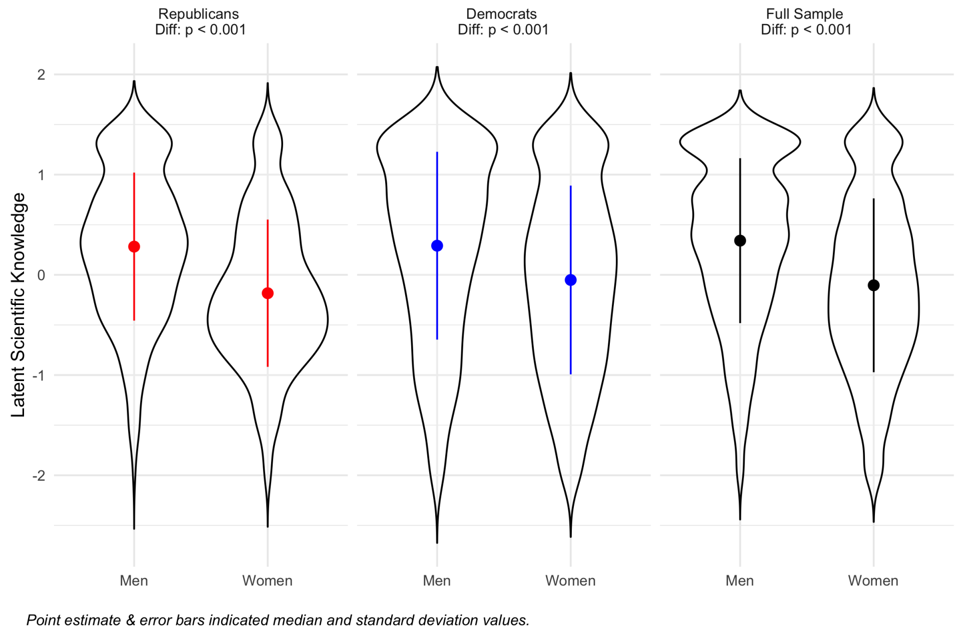

1.1 Model Variables Coding Schemes

Scientific Knowledge Questions: Taken from Pew `American National Trends Panel Survey` Wave 32 (January 7-21, 2019), we use the following questions to estimate latent scientific knowledge of respondents. Note that these questions are asked over a year prior to the onset of the COVID-19 pandemic in the United States, thus alleviating endogeneity concerns between pandemic conditions and attainment of scientific knowledge. Below we show the individual questions used to create our IRT measure of scientific knowledge, with the parenthesis indicating the percentage of respondents chose the response as an answer to the respective question.

1. Here's a different kind of question. (If you don't know the answer, select "Not sure.") As far as you know. . .

   - Fossil fuels (**Correct Answer**, 68%)
   - Biofuels (5%)
   - Geothermal resources (3%)
   - Renewable resources (6%)
   - Not sure (17%)
   - No answer (1%)

2. A scientist is conducting a study to determine how well a new medication treats ear infections. The scientist tells the participants to put 10 drops in their infected ear each day. After two weeks, all participants' ear infections had healed. Which of the following changes to the design of this study would most improve the ability to test if the new medication effectively treats ear infections?

   - Create a second group of participants with ear infections who do not use any ear drops (**Correct Answer**, 60%)
   - Create a second group of participants with ear infections who use 15 drops a day (5%)
   - Have participants use ear drops for only 1 week (13%)
   - Have participants put ear drops in both their infected ear and healthy ear (5%)
   - Not sure (16%)
   - No answer (1%)

3. Which of the following is an example of genetic engineering?

   - Inserting a gene into plants that makes them resistant to insects (**Correct Answer**, 56%)
   - Growing a whole plant from a single cell (6%)
   - Finding the sequences of bases in plant DNA (8%)
   - Attaching the root of one type of plant to the stem of another type of plant (9%)
   - Not sure (21%)
   - No answer (1%)

4. What is the main cause of seasons on the Earth?

   - The tilt of the Earth's axis in relation to the sun (**Correct Answer**, 63%)
   - The distance between the Earth and the sun (11%)
   - The speed that the Earth rotates around the sun (7%)
   - Changes in the amount of energy coming from the sun (4%)
   - Not sure (15%)
   - No answer (1%)

5. These graphs show the monthly precipitation and average temperature for three cities in the United States over the course of one year.

   - Chicago, Illinois (**Correct Answer**, 59%)
   - New York, New York (20%)
   - Los Angeles, California (9%)
   - They all have the same annual temperature range (3%)
   - Not sure (8%)

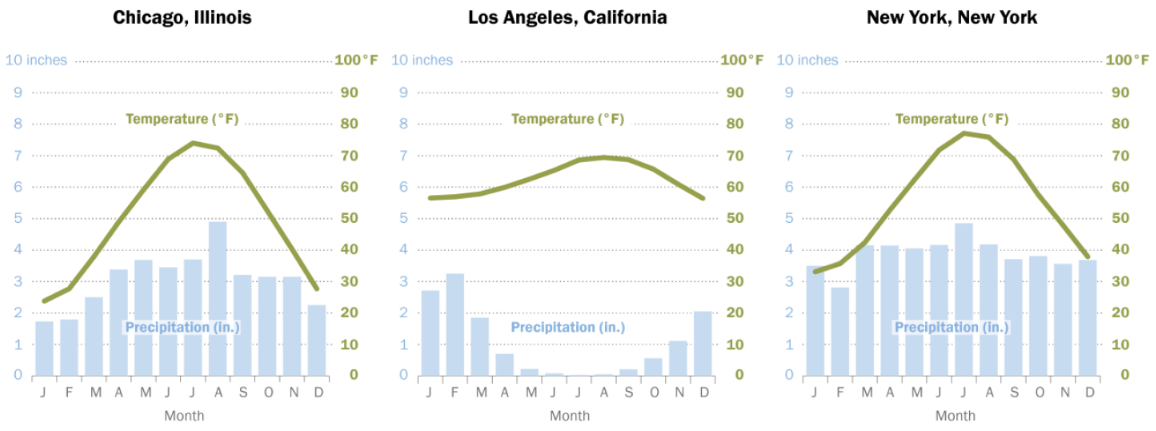

- No answer (1%)

6. The time a computer takes to start has increased dramatically. One possible explanation for this is that the computer is running out of memory.This explanation is a scientific. . .

   - Hypothesis (**Correct Answer**, 52%)
   - Conclusion (8%)
   - Experiment (4%)
   - Observation (19%)
   - Not sure (17%)
   - No answer (1%)

7. Many diseases have an incubation period. Which of the following best describes what an incubation period is?

   - The period during which someone has an infection, but is not showing symptoms (**Correct Answer**, 76%)
   - The recovery period after being sick (4%)
   - The effect of a disease on babies (2%)
   - The period during which someone builds up immunity to a disease (5%)
   - Not sure (12%)
   - No answer (1%)

8. When large areas of forest are removed so land can be converted for other uses, such as farming, which of the following occurs?

   - Increased erosion (**Correct Answer**,60%)
   - Colder temperature (3%)

- Decreased carbon dioxide (14%)
- Greater oxygen production (3%)
- Not sure (20%)
- No answer (1%)

9. An antacid relieves an overly acidic stomach because the main components of antacids are. . .
    - Bases (**Correct Answer**, 39%)
    - Acids (11%)
    - Neutral (13%)
    - Isotopes (3%)
    - Not sure (33%)
    - No answer (1%)
10. Which of these is a major concern about the overuse of antibiotics?
    - It can lead to antibiotic-resistant bacteria (**Correct Answer**, 79%)
    - There will be an antibiotic shortage (2%)
    - Antibiotics can cause secondary infections (5%)
    - Antibiotics will get into the water system (2%)
    - Not sure (11%)
    - No answer (1%)
11. A car travels at a constant speed of 40 miles per hour. How far does the car travel in 45 minutes?
    - 30 miles (**Correct Answer**, 57%)
    - 25 miles (4%)
    - 35 miles (14%)
    - 40 miles (9%)
    - Not sure (15%)
    - No answer (1%)

Gender: Pew binary measure coded 1 for women and 0 for men.

Self-Reported Partisanship: Nominal variable coded 1 for Republican, 2 for Independent, and 3 for Democrat.

Ideology: Self-identified symbolic ideology coded on a five-point ordinal scale from 1 (very conservative) to 5 (very liberal). A value of 3 indicates a self-identified moderate.

Age: Self-reported age variable coded on a 4-point ordinal scale in the following fashion: 1 (18-29), 2 (30-49), 3 (50-64) and 4 (65+).

Education: Self-reported highest educational attainment coded on a standard six-point ordinal scale in the following fashion: 1 (less than high school), 2 (high school graduate), 3 (some college), 4 (associate's degree), 5 (college degree), and 6 (post-graduate degree).

Race: Binary variable coded 1 if a respondent identifies as white non-Hispanic and 0 if the respondent identifies as otherwise.

Income: Self-reported income on a nine-point ordinal scale recorded in the following fashion: 1 (less than $ 10,000), 2 ($10,000-$20,000), 3 ($20,000-$30,000), 4 ($30,000-$40,000), 5 ($40,000-$50,000), 6 ($50,000-$75,000), 7 ($75,000-$100,000), 8 ($100,000-$150,000), and 9 ($150,000 or more).

Census Region: Contextual variable coded in a series of three dummy variables indicating residence in the Midwest, South, and West. The omitted baseline category of this variable is the Northeast.

1.2 IRT Model Specification

IRT models are a useful tool for measuring latent preferences or characteristics from a set of observed behaviors, with the canonical example being the measurement of students' abilities with multi-item tests. In this example, higher ability should correspond to a higher probability of a correct answer to a valid question. In political science, the IRT model has been applied to measure ideology, political knowledge, and other latent concepts from a set of observed indicators (Armstrong et al. 2014).

Figure 2
Item Characteristic Curves of Latent Knowledge & COVID-19 Policies

(a) Latent COVID-19 Restriction Policies (b) Latent Scientific Knowledge

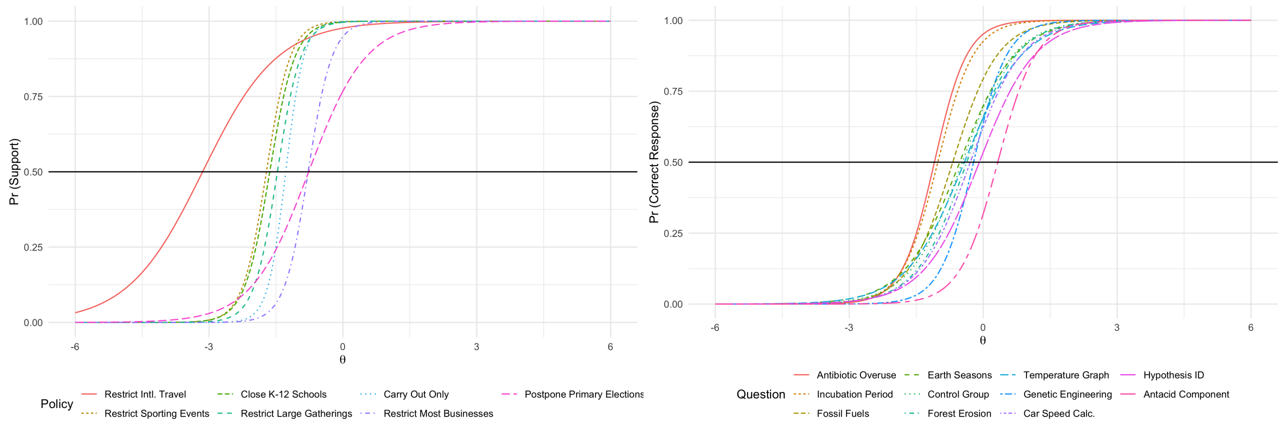

As the results of the IRT model show in Figure 2A, the greater degree of latent restriction attitudes the more likely an individual is to express that government intervention in limiting social activities is necessary for containing the spread of COVID-19. The 'hardest' policy to deem necessary was the postponement of state primary elections while the 'easiest' policy was restricting international travel.

To measure citizen scientific literacy, we rely on eleven questions asking panelists objective questions concerning scientific facts and concepts. These questions are designed to test scientific literacy across the life and physical sciences domains (Miller 1983; Kennedy & Hefferon 2019). Given inherent variation in difficulty across questions, we treat scientific knowledge as a latent variable and measure it using a graded scale item response theory model. Figure 2B shows the validity of our scientific knowledge measure by assessing the item characteristic curves of our composite measure. The x-axis articulates our measure of latent scientific knowledge and the y-axis articulates the probability of a current response for each of the questions assessing scientific knowledge.[1] As Figure 2B clearly shows, the greater the degree of latent knowledge the higher the probability of a correct response on each of the scientific knowledge questions, with clear variation in the difficulty of each question. For example, the easiest knowledge question to answer correctly concerned panelists ability to correctly identify that the overuse of antibiotics

1. Full scientific knowledge questions can be found earlier in the appendix.

can lead to antibiotic-resistant bacteria. By contrast, the hardest question to answer correctly dealt with identifying that the main components of antacids are bases.

References

Armstrong, Dave, Ryan Bakker, Royce Carroll, Christopher Hare, Keith T. Poole, & Howard Rosenthal. 2014. *Analyzing Spatial Models of Choice and Judgment with R.* Boca Raton, FL: Chapman / Hall/CRC.

Kennedy, Brian, & Meg Hefferon. 2019. "What Americans Know About Science." *Pew Research Center.*

Miller, Jon D. 1983. "Scientific Literacy: A Conceptual and Empirical Review." *Daedalus* 112 (2): 29–48.

Table 1

Full Sample: Interactive Effects of Scientific Knowledge & Gender by Policy

	Dependent Variable: Necessary Government Policy Measure							
	Restrict International Travel	Restrict Most Businesses	Restrict Large Gatherings	Restrict Sporting Events	Restrict K-12 Instruction	Restrict Restaurant Dining	Restrict Primary Election	Latent Restriction Scale
	(1)	(2)	(3)	(4)	(5)	(6)	(7)	(8)
Women Effect[1]	**0.031***	0.057	**0.051****	**0.101*****	**0.116*****	**0.051***	**-0.125*****	0.070
	(0.016)	(0.038)	(0.025)	(0.024)	(0.028)	(0.027)	(0.040)	(0.096)
Men Effect[1]	**-0.051*****	**-0.087****	-0.012	-0.003	0.014	0.036	**-0.133*****	**-0.172***
	(0.014)	(0.038)	(0.025)	(0.021)	(0.025)	(0.032)	(0.040)	(0.090)
Baseline Effect[2]	-0.014	-0.020	0.017	**0.044****	**0.060*****	**0.042****	**-0.128*****	-0.060
	(0.012)	(0.030)	(0.019)	(0.018)	(0.021)	(0.023)	(0.031)	(0.072)
Model Controls	✓	✓	✓	✓	✓	✓	✓	✓
Psuedo[1] R^2	0.66	0.63	0.66	0.66	0.64	0.63	0.62	0.057
Psuedo[2] R^2	0.66	0.63	0.66	0.65	0.64	0.63	0.62	0.056
AIC[1]	1,340.05	4,505.78	2,705.62	2,152.79	2,421.83	3,170.90	4,769.27	-
AIC [2]	1,352.56	4,510.89	2,707.60	2,171.67	2,433.32	3,169.58	4,767.28	-
N	2,775	2,775	2,775	2,775	2,775	2,775	2,775	2,775

Note: Min/Max Scientific Knowledge First-Difference Effect on Pr(Necessary Policy Support) Reported.

[1]estimated from interactive model, [2]estimated from additive model.

Latent restriction model (8) estimated via OLS. Models 1-7 estimated via logistic regression models.

$^{*}\rho <0.1$; $^{**}\rho <0.05$; $^{***}\rho <0.01$

Table 2

Democratic Partisans: Interactive Effects of Scientific Knowledge & Gender by Policy

	Dependent Variable: Necessary Government Policy Measure							
	Restrict International Travel	Restrict Most Businesses	Restrict Large Gatherings	Restrict Sporting Events	Restrict K-12 Instruction	Restrict Restaurant Dining	Restrict Primary Election	Latent Restriction Scale
	(1)	(2)	(3)	(4)	(5)	(6)	(7)	(8)
Women Effect[1]	-0.029	**0.221*****	**0.098*****	0.009	**0.069****	0.021	0.038	**0.272****
	(0.025)	(0.066)	(0.025)	(0.037)	(0.026)	(0.031)	(0.035)	(0.126)
Men Effect[1]	**0.101*****	0.039	-0.008	**-0.044***	**-0.039***	-0.021	**-0.143****	-0.172
	(0.027)	(0.054)	(0.027)	(0.025)	(0.022)	(0.031)	(0.064)	(0.120)
Baseline Effect[2]	**-0.066*****	**-0.126****	**0.046***	-0.020	0.013	-0.002	-0.059	0.032
	(0.021)	(0.030)	(0.051)	(0.026)	(0.022)	(0.022)	(0.027)	(0.101)
Model Controls	✓	✓	✓	✓	✓	✓	✓	✓
Psuedo[1] R^2	0.61	0.55	0.64	0.61	0.60	0.59	0.56	0.039
Psuedo[2] R^2	0.60	0.55	0.64	0.60	0.60	0.59	0.56	0.030
AIC[1]	487.62	1,235.61	578.60	529.86	610.49	694.53	1,490.95	-
AIC [2]	489.67	1,237.46	584.61	533.14	619.92	694.25	1,494.44	-
N	1,122	1,122	1,122	1,122	1,122	1,122	1,122	1,122

Note: Min/Max Scientific Knowledge First-Difference Effect on Pr(Necessary Policy Support) Reported.

[1]estimated from interactive model, [2]estimated from additive model.

Latent restriction model (8) estimated via OLS. Models 1-7 estimated via logistic regression models.

$^{*}\rho < 0.1$; $^{**}\rho < 0.05$; $^{***}\rho < 0.01$

Table 3

Republican Partisans: Interactive Effects of Scientific Knowledge & Gender by Policy

	Dependent Variable: Necessary Government Policy Measure							
	Restrict International Travel	Restrict Most Businesses	Restrict Large Gatherings	Restrict Sporting Events	Restrict K-12 Instruction	Restrict Restaurant Dining	Restrict Primary Election	Latent Restriction Scale
	(1)	(2)	(3)	(4)	(5)	(6)	(7)	(8)
Women Effect[1]	**0.289*****	0.014	0.011	**0.187*****	**0.252*****	**0.142****	**-0.147****	0.137
	(0.010)	(0.079)	(0.037)	(0.060)	(0.057)	(0.069)	(0.067)	(0.222)
Men Effect[1]	0.098	**-0.251*****	-0.034	-0.068	0.031	0.057	-0.005	-0.263
	(0.062)	(0.073)	(0.053)	(0.044)	(0.059)	(0.072)	(0.080)	(0.199)
Baseline Effect[2]	**0.156*****	**-0.134****	-0.015	0.039	**0.124*****	**0.092***	-0.067	-0.088
	(0.057)	(0.057)	(0.042)	(0.037)	(0.048)	(0.052)	(0.058)	(0.156)
Model Controls	✓	✓	✓	✓	✓	✓	✓	✓
Psuedo[1] R^2	0.58	0.56	0.58	0.60	0.59	0.55	0.55	0.072
Psuedo[2] R^2	0.57	0.56	0.58	0.59	0.59	0.55	0.55	0.070
AIC[1]	326.25	1,707.92	1,119.63	912.13	967.44	1,346.39	1,698.51	-
AIC[2]	330.14	1,710.73	1,117.75	927.35	975.41	1,345.74	1,698.38	-
N	783	783	783	783	783	783	783	783

Note: Min/Max Scientific Knowledge First-Difference Effect on Pr(Necessary Policy Support) Reported.

[1]estimated from interactive model, [2]estimated from additive model.

Latent restriction model (8) estimated via OLS. Models 1-7 estimated via logistic regression models.

$^{*}\rho < 0.1$; $^{**}\rho < 0.05$; $^{***}\rho < 0.01$
